# Standardization of gut microbiome analysis in sports

**DOI:** 10.1016/j.xcrm.2024.101759

**Published:** 2024-10-04

**Authors:** Laura Mancin, Antonio Paoli, Sara Berry, Javier T. Gonzalez, Adam J. Collins, Maria Antonia Lizarraga, Joao Felipe Mota, Segata Nicola, Ian Rollo

**Affiliations:** 1 Department of Biomedical Sciences, University of Padua, Padua, Italy; 2 Human Inspired Technology Research Center HIT, University of Padua, Padua, Italy; 3 Department of Nutritional Sciences, King’s College London, London, UK; 4 Department for Health, University of Bath, BA2 7AY Bath, UK; 5 Medical, Sport Science and Health Department, FC Barcelona, Barcelona, Spain; 6 APC Microbiome Ireland, Department of Medicine, School of Microbiology, University College Cork, T12 YT20 Cork, Ireland; 7 Centre for Integrative Biology, University of Trento, Trento, Italy; 8 Gatorade Sports Science Institute, PepsiCo Life Sciences, Global R&D, Leicestershire, UK; 9 School of Sports Exercise and Health Sciences, Loughborough University, Leicestershire, UK

**Keywords:** gut microbiome, metabolism, sport, exercise, methodology

## Abstract

The gut microbiome plays a significant role in physiological functions such as nutrient processing, vitamin production, inflammatory response, and immune modulation, which, in turn, are important contributors to athlete health and performance. To date, the interpretation, discussion, and visualization of microbiome results of athletes are challenging, due to a lack of standard parameters and reference data for collection and comparison. The purpose of this perspective piece is to provide researchers with an easy-to-understand framework for the collection, analysis, and data management related to the gut microbiome with a specific focus on athletic populations. In the absence of a consensus on microbiome research in the sports field, we hope that these considerations serve as foundational “best practice.” Adherence to these standard operating procedures will accelerate the path toward improving the quality of data and ultimately our understanding of the influence of the gut microbiome in sport settings.

## Teaser

This perspective article provides researchers with an easy-to-understand framework for the collection, analysis, and data management related to the gut microbiome in athletic populations. Adherence to these standard operating procedures will improve the quality of data and ultimately our understanding of the influence of the gut microbiome in sport settings.

## Introduction

Since 2005, advances in high-throughput sequencing technologies have allowed the analysis of microbiome DNA extracted directly from human stool samples.^1^ This technological progress spurred an increase in clinical studies profiling the gut microbiome across diverse populations, including healthy individuals,^2^ those with clinical conditions,^3^ and athletes.^4^ Notably, knowledge of the gut microbiome has become of particular interest to athletes,^5^ given its pivotal role in promoting health, facilitating nutrient processing, and influencing exercise performance.^6^

While metagenomics can contribute to our understanding of potential functions of the gut bacteria, it is crucial to emphasize the need for a rigorous and critical approach to investigate the metabolic interaction between the host and its gut microbiome. This approach is essential to prevent unrealistic expectations that could undermine the credibility of microbiome science and its clinical application. Therefore, well-controlled randomized controlled trials with different collection points favor these mechanistic and causal investigations.^5^^,^^7^

Despite the increasing interest and research efforts in gut microbiome studies among athletes, a standard protocol tailored to the characteristics of sport and exercise has yet to be established. Consequently, published human studies have varied in (1) clinical data collection, (2) stool sample collection techniques (whole fresh stool vs. dry swab), (3) timing and frequency for collection,^8^ (4) storage methods (−80°C, −20°C, +4°C vs. room temperature), (5) technical sample preparation (different DNA extraction protocols), (6) methods of microbial assessment (target gene vs. whole genome), (7) sequencing platforms (including Illumina HiSeq, PacBio RS II, Oxford Nanopore MinION), and (8) statistical analysis approaches.^9^ By standardizing these elements, more confident inferences can be made on gut microbiome results over time, as well as to facilitate comparisons between individuals within the same cohort.

Therefore, the aim of this paper is to provide guidelines to standardize the collection, analysis, and presentation of gut microbiome data in sport and exercise. We also provide a toolbox for researchers to be able to understand, interpret, and integrate microbiome analysis in their experimental and translational activities. We direct the readers to more specialized reviews on specific topics where these exist.

We complete our view with several practical examples of gut microbiome analysis strategies in the field of personalized nutrition and metabolomics in sport and exercise.

## Microbiome research in sport and exercise

In microbiome research, traditional scientific questions and principles for experimental design are typically the same as for any other experiment. Designing an experiment to generate meaningful data is a fundamental step discussed extensively in other reviews.^10^

In summary, we propose three major experimental frameworks for microbiome research.

First, “microbiome epidemiology,” which characterizes either microbial stability or temporal variability, may elucidate associations between microbiome variation and changes in athlete’s health status. Therefore, the combination of long-term follow-up analysis and advanced statistical approaches (i.e., mediation analysis or Mendelian randomization) may allow the comprehension of which bacterial features are temporally stable or show larger temporal variation and if this temporal variation can be linked to changes in the athletes’ clinical phenotypes (microbiome-phenotype associations).^11^

Second, randomized controlled trials (i.e., crossover trials) can be adopted to determine the causal interaction between a specific intervention, such as dietary intervention, and changes in athlete’s clinical parameters. For example, specific microbiome endpoints (i.e., compositional shifts, functions, microbially derived compounds) can be combined with surrogate markers of diseases (i.e., serum cholesterol level, glucose, blood count) and biological processes hypothesized to link metabolic activities of gut microbiome with the host (i.e., gut hormones, cytokines).^12^ Statistical analysis, such as regression analysis, allows to determine the potential mechanistic explanation between diet and athlete’s health. Of note, in the microbiome field, the choice of crossover design has advantages for randomized controlled trials (RCTs) as participants serve as their own controls, which allows for the removal of inter-individual variation of person-specific factors (i.e., baseline characteristics, genetics, microbiome). In this context, it is warranted to strictly control for confounding factors (Table 1) and consider habitual diet as the substantial confounder. However, controlled-feeding studies in free-living conditions, in which food are provided and recorded (i.e., elite athlete setting), may allow controlling for diet.^13^

**Table 1:** Athlete’s metadata collection in sport and exercise

**Demographics**	

Age (years)	
Gender	
Ethnicity (Asian, Black or African, Hispanic, White, Native-origin)	
Nationality	
Birth location	
Immigration history	
Environment: rural/urban	

**Medical history**	

Medical conditions: allergy, intolerances	
Birth type: vaginal, C-section	
Blood type: A, AB, O, unknown	
BMI (kg/m^2^), weight status	
Sleep (hours, quality)	
Circadian rhythm disruptions	
Menstrual cycle timing	
Menstrual cycle-related conditions (amenorrhea, polycystic ovary syndrome [PCOS], endometriosis, menopause status)	
Bowel habits: stooling frequency, timing	
Bristol stool scale	

**Diet**	

Diet quality (foods and nutrients), dietary habits (meal timing/fasting habits/meal ordering)	
FFQ (food frequency questionnaire), 24 h, 3 days food record, 24-h recall	
Intake of ready-to-eat meals? Yes, No	
Intake of sweeteners: yes, no	
Diet type (conventional/vegetarian/vegan/organic diet)	

**Intake of supplements or medicines**	

Antibiotic exposure previous weeks	
Alcohol consumption	
Dietary supplements: probiotics, prebiotics, melatonin, fibrate, among others	
Proton pump inhibitors	
Seasonal allergies medications	
Oral contraceptive	
Other drugs	

**Fitness data**	

Type of sport (strength, endurance, combat sport, team sport)	
Training frequency (no. of training per week, no. of competitions/games per week)	
Any kind of physiological data available: VO2Max, basal metabolism rate	

Third, longitudinal human interventions studies can be combined with specific statistical approaches (i.e., Bayesian networks or Mendelian randomization) to estimate causal effects among diet, microbiome, and athlete’s health^14^ and provide further points of intervention (e.g., with dietary guidelines or other methods of manipulating the gut microbiome).

However, completing intervention studies in elite athletes can be difficult due to the training and inflexible competition schedules.

**Figure 1:**
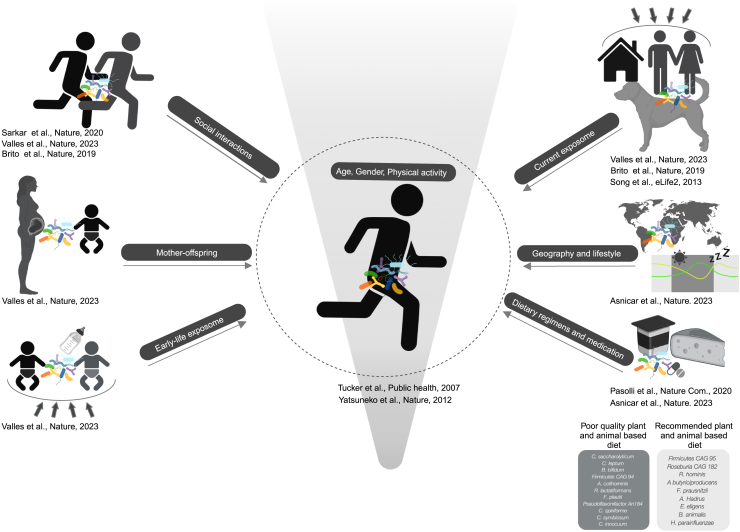
Factors associated with influencing the gut microbiome The genetic makeup of human gut microbiome is seeded at birth; however, the gut microbiome at strain level is shaped by a combination of extrinsic and intrinsic factors. Main drivers include genetics, dietary habits (plant and animal based diets), medications (nonsteroidal anti-inflammatory drugs, antibiotics, etc.), lifestyle, early-life exposures, childhood environment, adulthood exposome (neighborhood urbanicity and income), circadian rhythms, and geographical origin. Intrinsic factors include genetics and individual characteristics. In addition, the gut microbiome is shaped across a lifetime with the unique makeup of bacterial taxa acquired from other individuals (e.g., mother to offspring and social interactions transmissions). Created with BioRender.com.

Nonetheless, as many factors strongly influence the gut microbiome^15^ (Figure 1), it is imperative to accurately account for well-known confounders, such as biological factors, diet, exercise, lifestyle, and time frame of sample collection. Additionally, collecting athlete-specific metadata (as outlined in Table 1) is essential to enhance the analysis and the interpretation of results.

Standardizing logistical features (sample collection, transportation, storing) and technical factors (DNA extraction protocols, bioinformatic pipelines, software versions) also represent an essential recommendation to control for variation introduced by the intrinsic nature of the microbiome. This includes the inter-variability in microbial composition and functional metabolic responses.

## Data collection in sports environments

### Sampling collection

There is currently no consensus on the optimal method to collect stool samples for microbiome analysis. In short, the choice depends on feasibility, cost, population, environment, and computational methods that will be after used to read the microbiome downstream. In this paper, we present some methodological approaches aiming to minimize bias and enhance analysis resolution.

Sample collection, transportation, and preservation procedures represent the first crucial steps that ensure integrity and stability of the collected materia.^16^ Therefore, collection procedures and storage conditions must be optimized to prevent DNA degradation and standardized to reduce the variability. Key objectives are to collect a sufficient microbial biomass for sequencing and limit sample contamination.

Factors such as “length of time” between sample collection, “freezing method,”^17^ and the number of “freezes-thaw cycles” can significantly impact the microbial community profiles. As such, it is essential to collect, transport, and preserve all the samples following a standardized procedure. Importantly, all procedures used should be recorded and included as fundamental metadata, including (1) the time elapsed between the collection of stool and DNA extraction, (2) length of time passed in frozen storage, and (3) number of freeze-thaw cycles.

Several commercial collection kits are already available. Researchers and practitioners should consider the volume of sample necessary for the planned analysis,^18^ dependent on the research question, and the available resources. For example, swabs collection might not be ideal for studies requiring larger amounts of fecal material, such as those involving metabolomics analysis combined with sequencing.^19^

Currently, the “gold standard” protocol suggests the collection of fresh whole stool with a disposable stool collector or container (i.e., Fecontainer, Fe-Col, and ColOff to avoid specimen contamination) with immediate DNA extraction or freezing at −20°C/−80°C.^20^

However, this protocol may be impractical in sport setting due to limited resources and irregular sampling time points. In addition, the follow-up of many athletes would require at-home sampling, adding further variability to the time between sampling and freezing.

Based on these logistical challenges, other athlete-friendly alternatives are available in the field of sport. A convenient collection alternative is represented by dry swabs. This approach requires very little fecal material and allows participants to easily obtain the small amount of stool from a solid toilet paper/newspaper positioned into the toilet or from disposable stool collector. For its easy application, this method has been used in the American Gut microbiome project^21^ and metagenome-wide association studies.^22^

Although swabs collection approach is not appropriate for studies requiring a large fecal volume, such as for metabolomics analysis (∼10–30 g stool), it represents a valid alternative in the sport setting.

For these reasons, the most “suitable sample collection method” in sport settings is the scoop or spatula stool kit. With this approach, individuals can collect a few grams of fecal material into a small container using a sterile scoop or spatula, maintaining it at room temperature for 60 days. The advantages of this method are the following: (1) the scoop provides an adequate material for whole metagenomic analysis and metabolomics (without the need to collect whole stool) and (2) it allows individuals to store the stool sample at room temperature or in regular freezer boxes or common home freezer, limiting the challenges of “freezing method.” This would enable sample collection at training center or at home and without the need for urgent carriers for sample delivery or same-day DNA extraction requirements,^23^ minimizing sample handling and degradation.^24^

In summary, the microbial DNA quality of the “gold standard” is comparable to that of the “suitable sample collection method,” but the latter reduces unnecessary costs and challenges associated with transport, storage, and collection methodologies.

### Materials for sampling collection

#### Acquisition of whole stool samples

##### Consumables/equipment

(1) Sterile container for stool sample collection
(2) Collection hat (i.e., Fecontainer, Fe-Col, and ColOff) or a plastic clear container
(3) Sterile spatula or scoop
(4) Prelabeled biohazard bag (3″ × 5″) with absorbent pad (2″ × 3″)
(5) Small Therapak box or Clinical Pak for shipment

We recommend filling the athlete’s questionnaire for metadata collection for all the methods (Table 2).

**Table 2:** Strengths and weaknesses of sequencing methods commonly used to study microbial communities

Factors	16S rRNA	Shotgun metagenomics
Cost	Quick and less expensive (∼$ 50)	More expensive (∼$150–$250)
Sample preparation	Simple	Complex
Functional profiling	Only prediction of functional profile	It can directly reveal the relative abundance of microbial function genes
Taxonomic resolution	Limited to genus level	Genus, species, and strain level
Taxonomic coverage	Bacteria and Archaea	Bacteria, Archaea, viruses, plasmids, and microbial function genes
Sensitivity to host DNA	Low	High. Contamination can obscure microbial signatures, but this can be controlled by enhancing the sequencing depth
Biomass	Low (swab)	Medium-high (scoop or whole stool)
Characterization of novel members of microbiome	Yes (primers are universal)	It is possible with *de novo* assembly-based method
Integrative meta-omics	Yes	Shotgun can be integrated with mass spectrometry-based metaproteomic and metabolomic
Bias	PCR amplification bias, differences in primers and variable regions	No PCR related bias; potential experiment biases can be controlled by standardizing procedures from sampling to analysis
Existing large dataset	Yes	It has yet to reach the level of standardization characteristic of other
Bioinformatic skills	Beginner to intermediate	Advanced

rRNA, ribosomal RNA; PCR, polymerase chain reaction.

##### Collection instructions for participants

(1) Label the sample container with your name, date of birth, time, and date of collection (or apply the pre-printed label).
(2) Before collecting the stool sample, use an alcohol prep pads and put on a pair of disposable gloves.
(3) Place the collection hat in the toilet to catch the stool, such as a potty or an empty plastic food container, to ensure the collection of a clean sample (a sterile container should be used).
(4) Avoid urination while taking the sample, as it can damage the sample. Note: female athletes should collect their stool samples 5–7 days after their menstrual period to avoid blood contamination. It is also suggested to indicate the timing of their menstrual cycle at the time of sampling.
(5) Collect the stool sample, preferably during the first bowel movement of the day, using the collection spoon or wooden stick. Note: be careful not to contaminate the outside surface of the sample container.
(6) Collect all of the stool and place it into the sterile container and then stir/homogenize it with a sterile spatula for at least 1 min.
(7) Close the container tightly and place the container into a plastic biohazard bag.
(8) Maintain the biohazard bag on fridge (−4°).
(9) Place the biohazard bag into a box (i.e., FedEx Clinical Pak bag) and transport the samples as soon as possible (within 24 h) to the analytical laboratory.
(10) Throw away the collection hat and wooden stick.
(11) Wash your hands thoroughly with soap and warm running water and use an alcohol prep pads.

#### Acquisition of stool samples using the scoop/spatula method

##### Consumables/equipment

(1) Fecal collection tube (20 × 76 mm screw cap tube prefilled with DNA/RNA Shield [9 mL] and spoon attached to screw cap)
(2) Collection hat (i.e., Fecontainer, Fe-Col, and ColOff) or a plastic clear container or a clean newspaper
(3) Prelabeled biohazard bag (3″ × 5″) with absorbent pad (2″ × 3″)
(4) Small Therapak box (e.g., Fisher Scientific) or FedEx Clinical Pak for shipment

##### Collection instructions for participants

(1) Follow the same instructions as described in the collection instructions (steps 1 to 4) for whole stool samples.
(2) Do not spill the stabilizing liquid in the tube.
(3) Screw the cap of the fecal tube and use the sterile spoon or spatula provided to collect a small amount of fecal sample (0.5–1 g or 1 mL stool sample).
(4) Transfer the small fecal sample into the plastic tube.
(5) Pick up the cap and screw until tightly closed.
(6) Shake for 30 s to achieve homogenization of the fecal material with the preservatives liquid.
The sample can be stored at ambient temperature (4°C–25°C) for approximately 1 month before shipping. For longer periods of time, the sample must be frozen (−20°C/−80°C).
(7) Place the sample into a plastic biohazard bag (i.e., FedEx Clinical Pak bag) and transport the samples to the analytical laboratory.
(8) Throw away the collection hat and wooden stick.
(9) Wash your hands thoroughly with soap and warm running water and use an alcohol prep pads.

#### Acquisition of stool samples using the dry swab method

##### Consumables/equipment

(1) Swab collection tube (SafeCollect tube: 15 × 92 mm; cap: 19 × 15 mm; 2 mL DNA stabilizer solution; 30- or 80-mm breakpoint flocked swab).
(2) Collection hat (i.e., Fecontainer, Fe-Col, and ColOff) or a plastic clear container or a clean newspaper
(3) Prelabeled biohazard bag (3″ × 5″) with absorbent pad (2″ × 3″).
(4) Small Therapak box (e.g., Fisher Scientific) or FedEx Clinical Pak for shipment

##### Collection instructions for participants

(1) Follow the same instructions as described in the collection instructions (steps 1 to 4) for stool samples using the scoop/spatula method.
(2) Screw the cap of the fecal tube and use the swab provided to collect a minimum amount of fecal sample (no more than 200 mg of stool sample).
(3) Insert the swab tip into the microtube to transfer the small amount of fecal sample.
(4) Break the swab tip (breaking point 20–30 mm) leaving the swab tip in the collection tube (making sure that the swab is in contact with the solution).
(5) Replace the tube cap and screw until tightly closed.
(6) As the sample is stabilized, it should be stored under the same conditions as in the scoop/spatula method.
(7) Place the sample into a plastic biohazard bag (i.e.,: FedEx Clinical Pak bag) and transport the samples to the analytical laboratory.
(8) Throw away the collection hat and wooden stick.
(9) Wash your hands thoroughly with soap and warm running water and use an alcohol prep pads.

### Stool sampling frequency

As the number of longitudinal microbiome studies is increasing, controlling for within-individual variation becomes a fundamental consideration during study design. Given that the microbial ecology is intrinsically dynamic and varies throughout the day and from day to day,^25^ an important consideration is the timing and frequency of stool collection.

As an example, in studies examining the effect of diet (exposure) on the gut microbiome, it is advisable to analyze microbiome samples for at least 3 days post exposure or, at the very last, the day after exposure.^26^ The rationale behind this approach is to ascertain how long it takes for a given exposure (such as dietary changes) to impact the gut microbiome composition.^8^

For this reason, we recommend to standardize the collection by (1) collecting the first full bowel movement of the day^27^ and (2) obtaining multiple consecutive microbiome samples per study time point (i.e., three daily sequential fecal samples in aggregate for diet-microbiome study).^25^

Repeated sampling of the gut microbiome allows to detect microbiome differences by mitigating the “within-person” bias, thereby reducing the need to increase sample size. Additionally, it aids in establishing causality and facilitates machine learning and mediation analysis.

### Stool sample transporting and storage

Although there is bias involved when using fecal samples as a proxy for intestinal microbial profiles, this method remains frequently used in human research studies due to its natural sample occurrence, repeatability, and non-invasive nature.^28^ While fecal sampling may appear a relatively simple method of gut microbiome assessment, there are several considerations and confounding factors that should be controlled for.

When fresh samples cannot be analyzed immediately, the widely regarded gold standard is to freeze samples instantly at −80°C, since this approach maintains microbial integrity relative to fresh samples.^29^ However, this approach would likely require samples to be collected within a short time window, which may not always be possible due to natural variations in bowel movement timing within and between individuals. In such cases, an alternative method should be implemented to reduce systematic bias in preprocessing steps.^30^

Several processing methods have been compared to ultra-low freezing as a reference, including refrigeration at 4°C, storage at room temperature, freezing in domestic appliances at −20°C, and the use of preservatives, as well as the effect of storage time. Gratton et al.^31^ recommended that when immediate ultra-low freezing is not possible, samples should be collected and kept at 4°C during storage and transportation since this method resulted in fewer microbial and metabolic profile changes than both room temperature and −20°C storage. In addition, the authors recommended that samples kept at 4°C should be processed ideally within 1 h of collection, and a maximum of 24 h, a finding further supported by Penington et al.^32^ Furthermore, Choo et al.^33^ found no significant differences in fecal microbiota diversity or composition of samples stored at 4°C compared to −80°C control samples, whereas storage at room temperature and the use of preservatives resulted in significant sample disturbances.

For remote participants where short-term (i.e., <24 h) storage is impractical, the use of a DNA stabilization buffer may be the most appropriate method of storage and transportation. Several commercially available kits with a stabilization buffer are available, and studies have compared the efficacy of these kits over time. One study observed that relative to −80°C control samples, OMNIgene.GUT displayed the least sample deviation after 72 h compared to other commercially available kits.^33^ Others have shown that fast technology analysis (FTA) cards and OMNIgene.GUT resulted in fewer compositional changes over 8 weeks compared to RNAlater, and preservation in 95% and 70% ethanol.^34^

In summary, the method selected to transport and store fecal samples should aim to minimize deviations in sample integrity, while considering the feasibility of each available method. In addition, the method chosen should be standardized across samples to reduce potential confounding variations.

## Sample analysis in the lab

### Data preparation

The appropriate type of experimental analyses, which includes DNA extraction, sequencing methods, and statistical analyses, is dependent on the scientific aim of the experiment. The detailed description of each experimental method is beyond the scope of this paper; nevertheless, we aim to provide evidence of the substantial technical variability that exists among different methods to encourage researchers to adopt a standardized approach. Therefore, the standardization of the processes, from experimental designing to sample storage, is necessary to obtain trustable results and potential translatability from lab-scale microbiome studies to clinical application.

### DNA extraction

It is important to note that DNA extraction is very susceptible to bias, significantly impacting the analysis results. Given that the substantial technical variability among different DNA extraction methods is relatively high, we generally recommend using the same reagent kits for all the samples in a study.^35^ The choice of DNA extraction method following sampling and storage has an impact on the revealed microbial community composition.^36^

Experimental studies comparing DNA extraction methods in stool samples (such as conventional or quantitative polymerase chain reaction (PCR), bands on denaturing gradient gel electrophoresis, phylogenetic microarray) showed significant differences in microbial relative abundance assessed by 16S rRNA sequencing.^37^ For instance, the first step of DNA extraction—disruption and/or lysis of the bacterial membranes—can introduce bias in evaluating specific bacterial taxa due to differences in cell wall structure (i.e., differences between gram-positive and gram-negative bacteria). Generally, DNA extraction methods that involve mechanical lysis/bead beating (mechanical disruption of the bacteria) are often considered superior to those that rely on chemical lysis.^38^ However, DNA extraction kits adopting the same bead-beating method have reported significant differences in DNA yield and bacterial DNA composition,^39^ highlighting the importance of standardization (using the same DNA extraction kit) for all samples.

In line with this context, the use of the same DNA extraction kit along with blanks^40^ (artificial samples designed to monitor the introduction of artifacts into the analytical process) should be adopted to avoid the potential introduction of contaminating microbial DNA during sample preparation, especially for low-biomass samples (i.e., stool collected with swabs). Additionally, the use of mock communities—reference samples with a known composition—and as well as the use of same standard specimens in each DNA sequencing run may be useful to attain the highest accuracy during analysis.

To mitigate differences between DNA extraction techniques, we advise centralizing the DNA extraction process for an experiment (the same laboratory) and employing the same extraction kits for all the samples. However, given that the same kit used twice can yield different results for the same samples,^41^ we additionally recommend to include balanced cases and controls in each batch of DNA extractions and accurately report metadata (including the DNA extraction batch information for each sample).

### Sequencing methods

Different approaches for investigating microbial communities can produce different results, and each method has strengths and weakness. Thus, the choice of the sequencing method/type relies on the scientific question, hypothesis, and analysis goals. Two common types of protocols are marker gene and shotgun metagenomic sequencing.

Marker gene analysis incorporates primers that target a specific region, such as 16S rRNA for bacteria and archea and internal transcribed spacer for fungi, in order to provide microbial phylogenies of a sample. Amplicon sequencing is cost-effective and applicable to low-biomass specimens contaminated by host DNA. However, it is limited to genus-level taxonomic resolution and is susceptible to inherent biases such as the number of PCR cycles^42^ and variable region selection and amplicon size.^43^

Whole metagenomic analysis involves sequencing all DNA present in a sample, including bacterial, viral, eukaryotic, and host DNA. Given adequate sequencing depth (the number of times that a given nucleotide in the genome has been read), it extends taxonomic resolution to species or strain level and profiles the functional capacity of a microbial community at the gene level.^44^ For a comprehensive review on metagenomics, we direct readers to the refeerence by Quince and colleagues.^45^

Marker gene sequences is often used to gain a low-resolution overview of a microbial community composition, typically applicable to large-scale studies. Alternatively, to gain strain-level resolution and microbial functional analysis, whole metagenomic sequencing should be the first choice^46^^,^^47^ (Table 2).

Given that strain-level variants are crucial in determining gut microbial functional capacities, including interaction with host tissues, modulation of immune homeostasis, and xenobiotic metabolism,^48^^,^^49^ in elite sport environment, where the main goal is to understand how microbiome interacts with host physiology (e.g., functional capacity of microbiome in influencing athlete’s health and performance), we recommend whole metagenome sequencing. Despite being more expensive, the cost of shotgun metagenomics is reducing rapidly.^5^

### Library preparation and sequencing

Before DNA samples can be read by sequence, they must be fragmented, end-repaired, and collected into adapter-ligated libraries, in a multi-step process called “library preparation.” The choice of library preparation depends on the availability of materials, services, cost, and quantities of DNA. Similar to DNA extraction methods, standardizing the library preparation method for all samples within an experiment is crucial, as it significantly impacts metagenomic shotgun sequencing data.^40^

Currently, the Illumina platform is predominantly used in shotgun metagenomics due to its high outputs (>1.5 Tb per run) and high accuracy (∼0.1%–1% error rate). However, innovative long-read sequencing technologies (e.g., Oxford Nanopore MinION and Pacific Biosciences Sequel), which can generate up to 10 Gb per run, are being used in the latest metagenomic studies.

Multiple approaches are available for the generation of Illumina sequencing libraries, and the differences between methods rely on the type of fragmentation used. For example, transposase-based “tagmentation” method,^50^ as used in the Illumina Nextera XT, is popular in metagenomics given its low cost ($25–$40 for each sample) and minimal DNA requirement (∼1 ng DNA, but smaller amount can be used). However, it requires the subsequent PCR amplification step. Conversely, PCR-free systems which rely on physical fragmentation (e.g., KAPA HyperPrep PCR-free and PCR-free TruSeq DNA) may be preferred for their ability to reduce the biases from PCR amplification.^51^ Stool specimens often yield sufficient material for PCR-free system (∼250–500 ng of DNA). Therefore, we recommend choosing PCR-free-based methods given the ability to minimize PCR bias in calculations of abundance and improving assemblies for accurate taxonomic assignment.

Lastly, choosing the appropriate sequencing systems to maximize the output and the read length (the number of base pairs sequenced from a DNA fragment) depends on factors such as sample type, application, and coverage requirements. However, no published guidelines exist for the “correct” amount of coverage. Illumina HiSeq 2500 or 4000, NextSeq, and NovaSeq produce high volume of sequencing data (from 120 Gb to 1.5 Tb per run) and can be easily adopted in metagenomic studies. Researchers can decide the level of multiplexing (pool and sequence together DNA fragments from different samples to increase sample throughput) and set the sequencing depth (ultra-deep sequencing or shallow sequencing^52^) per sample. Generally, the Illumina platforms differ in their total output; for example, Illumina HiSeq 2500, which is the most popular choice in shotgun metagenomics, can quickly generates 2 × 250-nt reads in “rapid-run mode” or up to 1 Tb in high-output mode, with 2 × 125-nt reads (typically used for larger studies or when the greatest depth of coverage is required). The recently released NovaSeq Sequencing 6000 platform can generate outputs of up to 6 Tb and 20 B single reads in less than 2 days; however, the machine itself is costly (∼$985,000).

Independently from the platform choice, we encourage the use of “unique dual indexing” barcode for sequencing on newer Illumina machines in order to mitigate the barcode index hopping, a specific cause of index misassignment.

## Post-processing data analysis

### Bioinformatics analyses: From raw data to taxonomy and functional profile

Bioinformatics is an interdisciplinary field of science that combines biology, computer science, mathematics, and statistics to analyze and interpret biological and clinical data.

After completing the sequencing step, the raw sequencing files will be generated in FASTQ format (standard format for storing the output of high-throughput sequencing instruments such as the Illumina sequencers platforms). From here, there are several steps required to process the sequenced data before conducting analysis and generating reports.

Computational analysis aims to determine the taxonomic content (which organisms are present), and to estimate the functional capacity of the sample (which genes are present). This can be addressed by assigning sequencing reads to taxa and functional categories, based on their alignments to a reference database, in a process called binning. However, computational analysis performed on the data after sequencing depends on the type of sequencing performed.

For marker gene analyses, one common strategy is to cluster similar sequencing into operation taxonomic units (OTUs) using a 97% similarity threshold,^53^ known as “OTU picking” process. However, this method may miss subtle real biological sequence variation, such as single nucleotide polymorphisms. Oligotyping, which identifies the diversity of closely related but distinct bacterial organisms, can improve traditional OTU picking since it includes position-specific information from 16S rRNA sequencing. Some other amplicon-specific error-correction algorithms such as DADA2^54^ and Deblur algorithms^55^ may replace OTU-based approaches since such methods infer sample sequences exactly and resolve differences of as little as 1 nucleotide. To date, the use of amplicon sequence variant-based analyses, such as through DADA2, represents the gold standard for 16S analysis.

The next key step involves the assignment of the taxonomic name to each microbial sequence. Taxonomic assignments generally are performed using naive Bayesian classifiers such as the Ribosomal Database Project (RDP) classifier^56^ against reference databases such as Greengenes, SILVA, and RDP, depending on the amplicon target. It should be ensured that the most recent version of these databases is used.^57^

The most popular microbiome analysis package such as QIIME 2^53^ and mothur^58^ can provide support for taxonomic classification. Finally, a predictive functional profiling can be implemented^59^ to predict metagenomic content and thus the putative biological function of a microbial community. However, insights into the functional information of gut microbial community vanish when restricting the analysis to taxonomic assignment of 16S rRNA data.^60^

Alternatively, metagenomic analyses can directly infer the relative abundance of microbial functional genes and provide attainable taxonomic and phylogenetic identity at species and strains levels.^61^ Numerous approaches for metagenomics analyses have been published, and choosing “the best” is a demanding task and depends largely on the aim of the study.

Typically, metagenomics first requires a preprocessing step to remove either host DNA or rRNA and host RNA. Then, the pre-processed sequences can be analyzed by read-based profiling such as Kraken,^62^ MEGAN,^63^ and HUMAnN3.0^44^ to generate taxonomic or functional profiles or by assembly-based analyses such as MEGAHIT.^64^ To note, as for taxonomy and functional annotations, the choice of database is fundamental, since the taxonomic or functional assignments rely upon homology between the single reads and a reference. For example, curated genome databases for human gut samples include RefSeq,^65^ UniRef,^66^ and MetaHIT.^67^

Another computational approach for analyzing metagenome sequencing reads is *de novo* assembly. Metagenome *de novo* assembly is conceptually similar to whole-genome assembly^68^ and allows to assemble several short reads into longer sequences by breaking each sequencing read into overlapping sub sequences (fragments of DNA) of a fixed length *k* (contigs). These contigs can then be assembled by similarity to reconstruct partial or full genomes of microbes. Although this method may provide a more complete picture of the microbial community, metagenomic assembly presents several challenges and it is not universally applicable (it cannot be suitable for low-abundance genomes or in presence of different strains within the same bacterial species). For a comprehensive view of the *de novo* assembly approach, see references of Quince et al.^45^ and Vollmer et al.^69^ We highly recommend standardizing the molecular work and bioinformatic processing to generate appropriate results and basic summary reports as adopted in the American Gut Project.^21^

However, regardless of the sequencing technologies adopted and computational analysis performed, the complex nature of microbiome data requires an additional preprocessing step to improve data quality in the early stages of analysis. Preprocessing analysis may include many different techniques, but we provide the typical and standard workflow which includes quality control check, batch effect correction, and removal of contamination. Quality control represents the initial step where specific sequencing adapters and bases with a low base calling score (Phred score) are trimmed from the sequencing reads. Low-quality reads, adapter sequences, and bases that fall below a certain quality threshold are removed. FastQC (https://www.bioinformatics.babraham.ac.uk/projects/fastqc/) and Trimmomatic^70^ are generally used for this analysis.

Batch effects refer to systematic variations that may occur during data acquisition or processing, such as technical artifacts or biological factors. These effects may bias the results by introducing spurious variations in a group of samples. To address batch effects, careful study designs with balanced sample sizes across groups, normalization, and appropriate statistical models should be adopted. Considering batch effects may improve the reliability and reproducibility of results.^14^ Batch effects can be removed by raw data with methods such as univariate ComBat,^71^; alternatively, methods including Bayesian Dirichlet-multinomial regression meta-analysis^72^ can estimate unknown batch variables and incorporate them as covariates in linear models. Lastly, it is fundamental to detect and remove contamination (e.g., exogenous DNA) introduced externally or internally. Inclusion of negative and positive control samples is the recommended way to measure, detect, and mitigate contamination. To overcome these issues, different contamination detection tools such as Squeegee^73^ and MicrobIEM^74^ should be considered.

### Higher-level analysis to summarize data

Regardless the methods used for primary sequencing analyses, the output typically generates a matrix representing features abundance (i.e., species, taxa, genes). This output is quite “noisy” since microbiome data are highly dimensional, with many zero present in the matrix, and thus it requires appropriate statistical methods to interpret the matrices and decipher how the results correlate with the sample metadata.

Post-processing tools generally include traditional supervised methods, such as multivariate statistical analysis (i.e., ANOVA) and machine learning techniques. Machine learning methods such as clustering and correlation analyses with visualization tools (i.e., random forest, heatmap, and ordination, including principal-component analysis [PCA] and principal coordinate analysis [PCoA]) allow microbiome data to be graphically revealed.

Another approach to summarize data is by calculating diversity, including both alpha and beta diversity.

Alpha diversity is a measure of diversity within a sample or environment and can be easily compared across different sample groups. Different methods are available for calculating alpha diversity. Some measures, including *Chao1* abundance estimator and *Faith’s phylogenetic distance*, are adopted to calculate the “richness” (the number of OTUs or species present in a sample), while *Shannon index* and *Simpsons diversity index* are common methods for combining both “richness” and “evenness.”^75^

Beta diversity measures the distance (or dissimilarity) between each pair of samples and generates a distance matrix. Various metrics have been developed to calculate beta diversity, and each has its own advantages and disadvantages. The most common beta diversity metrics are quantitative metrics such as Bray-Curtis (non-phylogeny-based method that takes abundance into account) and weighted UniFrac (that uses the abundance information for each OTU along with their phylogenetic distances), as well as qualitative metrics such as un-weighted UniFrac (which uses the presence and absence of OTUs between samples along with their phylogenetic distances).

The software used to calculate alpha and beta diversity include QIIME 2,^53^ mothur,^58^ and the R package vegan (VEGAN, a package of R functions for community ecology). For testing significant beta diversity clustering between different groups, robust statistical testing such as the non-parametric permutation test (Permutational Multivariate Analysis of Variance [PERMANOVA]) is usually adopted.^76^ Visualizing beta diversity data can be obtained using *ad hoc* graphical representation methods such as PCoA or PCA, which reduce large and complex distance matrices into visually manageable two-dimensional or three-dimensional plots. These plots, known as PCoA plots, intuitively reveal clustering patterns within the samples and can be colored by various metadata categories for better visualization. Another common analysis method is to examine differential abundant species in the comparison groups of interest (e.g., interventions vs. controls).

### Data visualization

In marker gene studies, standard bioinformatic reporting includes plots illustrating taxonomic composition, alpha diversity, beta diversity (ordination), differential abundance testing, and multivariate analysis (e.g., PERMANOVA). These plots are complemented by raw bioinformatics outputs (OTU table, taxonomic classification of each OTU), differential abundance results, and relative abundances of taxa at various taxonomic levels.

For shotgun metagenome sequencing high-resolution taxonomic and functional profiles are analyzed observing the same principles that guide the analysis of amplicons. The main metagenomics pipeline is based on tools developed to characterize human microbiomes, specifically HUMAnN3.0, MetaPhlAn 4.0, and StrainPhlAn 4.0 from The Huttenhower Lab (https://github.com/biobakery/biobakery/wiki/).

## Microbial functionality and metabolomics: Integrating omics in sport and exercise

The relative bacterial composition and diversity of fecal samples (a proxy for the gut microbiome) are often assessed using 16s rDNA gene sequencing. However, these techniques can only determine which bacteria are present and thus cannot infer functional capacity of the microbiota.^77^ To overcome this limitation, numerous studies are beginning to use a meta-omics approach to elucidate the physiological impact of the microbiota and its integrative processes. For instance, metagenomics investigates the gene content from uncultivated microbes and can determine the function of these microbes, i.e., “what are they able to do?”. Additionally, metabolomics can characterize metabolite profiles, i.e., “which molecules are there.”^77^

A recent study exemplifies the importance of using an integrated meta-omics approach. Grosicki et al.^78^ collected stool samples from 12 triathletes to characterize acute changes in the fecal microbiome and metabolome following an ultra-endurance triathlon. Compared to pre-race, post-race fecal bacterial taxa remained stable, despite significant changes in fecal metabolite concentrations, such as fatty acids and bile acids. Further, Barton et al.^25^ showed that when comparing professional male rugby players with non-athlete controls, there was a greater separation between the groups at the metagenomic and metabolomic levels than there was at compositional levels. These studies highlight the importance of an integrated approach considering the observed independent shifts within the gut microbiome.

The functional impact of certain bacterial taxa and fecal metabolites appears to be linked with athletic performance and should therefore be considered equally important as microbial composition when designing microbiome studies. Indeed, Barton et al.^25^ showed that the gut microbiomes of athletes tended to exhibit higher abundance of fecal metabolites (e.g., short chain fatty acids [SCFAs]) compared to those of less active individuals. Notably, these metabolites, specifically butyrate, have been associated with cardiorespiratory fitness.^79^ The bacterial species *Veillonella atypica* was observed to be relatively abundant in marathon runners post-race. Interestingly, this study also showed that inoculating this strain of bacteria into mice resulted in greater exhaustive treadmill run time.^80^ Scheiman and colleagues speculated this performance benefit was mediated by the metabolic conversion of lactate to propionate, although others have argued that the choice of control confounded these results.^81^ Regardless, the potential impact microbial and metabolite function may have on performance highlights the importance of integrating meta-omics into sport and exercise research.

## Microbiome and personalized nutrition

Distinct bacterial species in the gut microbiome strongly correlates with favorable and unfavorable markers of cardiometabolic health,^82^ as well as dietary and lifestyle exposures.^83^ Despite being a dynamic entity, the gut microbiome is characterized by significant intra- and inter-person variation. The influence of host genetics on species composition is limited^84^ and estimated to contribute to around 5%^82^ of microbial composition. The ZOE PREDICT 1 study which explored inter-individual variations in microbiome and associations with a breadth of diet and lifestyle exposures and health measures in 1,098 individuals showed that identical twins share 34%^82^ of microbes at the species level, while unrelated individuals share 30%, underscoring the highly personalized nature of the gut microbiome, where non-genetic, modifiable factors, such as diet, are major contributors.

Health is a prerequisite for athletic performance. Strong correlations between dietary patterns and microbiome composition exist and may underpin some of the intra- and inter-person variation. Plant-based foods ingestion, classified by the healthy plant-based diet index hPDI, the healthy food diversity index, and the alternate Mediterranean diet score, all of which are associated with reduced risk of chronic disease,^82^ demonstrates a significant correlation with microbial composition.^82^ Modifications in diet can lead to rapid changes in gut microbial composition within days.^85^ The evidence linking gut microbial composition to a wide range of health measures is also gaining momentum. Associations have been reported between gut microbial species and cardiometabolic, gut, and mental health as well as cancer and many other non-communicable diseases. Evidence is now also emerging that dietary modifications which modulate microbial composition elicit downstream impacts on health outcomes.^86^ However, further research is required to establish direct causal effects of diet on microbiome-mediated health outcomes before we can confidently modify specific species to elicit changes in specific health outcomes. Furthermore, the personalized interventions created to date are based on associations studies, which may not imply a real microbiome-mediated effect.

Given the evidence linking diet-microbiome-health interactions and the highly personalized nature of the gut microbiome, personalized nutrition involving microbiome-informed guidance is a growing area of research and commercialization.^87^ Personalized nutrition leverages human individuality to drive nutrition strategies that prevent, manage, and treat disease and optimize health.^88^ It should be noted, however, that often personalized nutrition approaches are premature, without establishing whether there is true inter-individual heterogeneity of response to a nutritional intervention. Establishing the participant-by-treatment interaction using a repeated period (replicate) crossover trial can provide the evidence for inter-individual heterogeneity of response, and therefore they indicate the potential or futility of identifying responders.^89^

Human individuality is shaped by biological features (i.e., metabolome and microbiome composition) as well as lifestyle, the way we eat our food (i.e., fasting period and meal ordering), and the reason we make the dietary choices. Therefore, the optimal framework for personalized athlete nutrition will be one that accurately captures many features of human individuality (between and within individuals) as well as our individual gut microbiome profiles.

Studies have demonstrated significant inter- and intra-individual variations in glycemic^90^ and lipemic control,^91^ as well as gut microbiome composition, highlighting the need to conduct studies that repeatedly test multiple nutritional treatments in the same person, including the same treatments multiple times (comparing each individual at least twice). Employing the use of artificial intelligence algorithms affords the field of personalized nutrition the ability to predict individual responses to dietary interventions, incorporating genetics, gut microbiome, and other clinical and lifestyle characteristics. Recent research demonstrates the potential of personalized approaches integrating microbiome composition to improve health outcomes in favor of generic population-based guidelines.^92^ Emerging evidence also supports the notion that personalized nutrition advice is associated with improved adherence,^93^ paving the way for novel, targeted dietary interventions to modulate the gut microbiome and, by extension, athlete health.

## Conclusion

**Figure 2:**
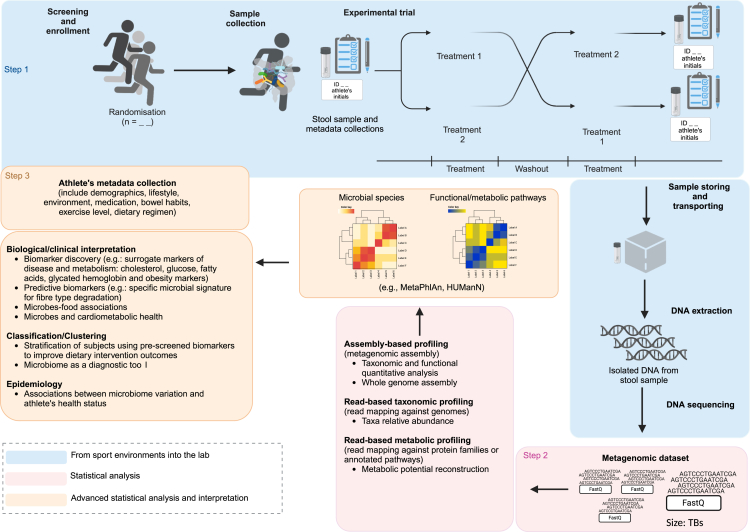
Schematic overview of “best practices for microbiome research” in the field of sport and exercise Step 1: into the field and into the lab. Most appropriate methods for study design. Step 2: statistical analyses. Computational analyses are performed to determine the taxonomic content (which organisms are present within the sample) and the functional capacity of the sample (which genes are present). Analyses can include both read-based and assembly-based approaches depending on the experimental design. Read-based metabolic profiling analysis can be also included. Step 3: advanced statistical analysis and data interpretation. Different advanced statistical methods can be used to interpret the data. In this case, it could be useful to discover potential correlation between markers of disease or metabolic health and specific bacterial taxa (at strain level). Association between microbiome variation and athlete’s phenotype variation can be also identified with longitudinal and epidemiological studies. Created with BioRender.com.

In conclusion, the standardization of microbiome analysis allows researchers and practitioners to make confident inferences on gut microbiome results over time as well as to facilitate comparisons between individuals within the same cohort. The current paper introduces a framework to inform the collection, analysis, and data management related to the gut microbiome (Figure 2). The adherence to these standard operating procedures will accelerate the path toward improving the quality of data and ultimately our understanding on the influence of the gut microbiome in sport settings.

## Declaration of interests

R.I. is an employee of the Gatorade Sports Science Institute, a division of PepsiCo, Incorporated. The views expressed in this article are those of the authors and do not necessarily reflect the position or policy of PepsiCo, Incorporated.
